# Small molecule glucagon release inhibitors with activity in human islets

**DOI:** 10.3389/fendo.2023.1114799

**Published:** 2023-04-19

**Authors:** Michael A. Kalwat, Karina Rodrigues-dos-Santos, Derk D. Binns, Shuguang Wei, Anwu Zhou, Matthew R. Evans, Bruce A. Posner, Michael G. Roth, Melanie H. Cobb

**Affiliations:** ^1^ Lilly Diabetes Center of Excellence, Indiana Biosciences Research Institute, Indianapolis, IN, United States; ^2^ Department of Pharmacology, University of Texas Southwestern Medical Center, Dallas, TX, United States; ^3^ Department Biochemistry, University of Texas Southwestern Medical Center, Dallas, TX, United States; ^4^ Indiana University School of Medicine, Center for Diabetes and Metabolic Diseases, Indianapolis, IN, United States

**Keywords:** glucagon, alpha cell, chemical biology, high-throughput (HT) screening, Type 1 diabetes (T1D)

## Abstract

**Purpose:**

Type 1 diabetes (T1D) accounts for an estimated 5% of all diabetes in the United States, afflicting over 1.25 million individuals. Maintaining long-term blood glucose control is the major goal for individuals with T1D. In T1D, insulin-secreting pancreatic islet β-cells are destroyed by the immune system, but glucagon-secreting islet α-cells survive. These remaining α-cells no longer respond properly to fluctuating blood glucose concentrations. Dysregulated α-cell function contributes to hyper- and hypoglycemia which can lead to macrovascular and microvascular complications. To this end, we sought to discover small molecules that suppress α-cell function for their potential as preclinical candidate compounds. Prior high-throughput screening identified a set of glucagon-suppressing compounds using a rodent α-cell line model, but these compounds were not validated in human systems.

**Results:**

Here, we dissociated and replated primary human islet cells and exposed them to 24 h treatment with this set of candidate glucagon-suppressing compounds. Glucagon accumulation in the medium was measured and we determined that compounds SW049164 and SW088799 exhibited significant activity. Candidate compounds were also counter-screened in our InsGLuc-MIN6 β-cell insulin secretion reporter assay. SW049164 and SW088799 had minimal impact on insulin release after a 24 h exposure. To further validate these hits, we treated intact human islets with a selection of the top candidates for 24 h. SW049164 and SW088799 significantly inhibited glucagon release into the medium without significantly altering whole islet glucagon or insulin content. In concentration-response curves SW088799 exhibited significant inhibition of glucagon release with an IC50 of 1.26 µM.

**Conclusion:**

Given the set of tested candidates were all top hits from the primary screen in rodent α-cells, this suggests some conservation of mechanism of action between human and rodents, at least for SW088799. Future structure-activity relationship studies of SW088799 may aid in elucidating its protein target(s) or enable its use as a tool compound to suppress α-cell activity *in vitro*.

## 1 Introduction

Type 1 diabetes (T1D) is not only a disease of the β-cell, but also of the glucagon-secreting α-cell. Glucagon is an important contributor in T1D pathogenesis (1). Over-secretion of glucagon by α-cells contributes to hyperglycemia in diabetes (2), and can even present during early stages of islet autoimmunity before β-cells are destroyed (3). Blockade of glucagon secretion from α-cells or glucagon action at its receptor as a T1D therapy is an active area of research, and this concept has led to the pursuit of potential therapies that prevent glucagon secretion and action (4–6). For example, recent work indicates beneficial effects of injected anti-glucagon receptor antibodies in humans with T1D (7) as well as non-human primates (8) and rodents (9). However, another approach is to normalize α-cell function, as opposed to completely eliminating glucagon secretion. While it is becoming clear that targeting glucagon may allow for new effective therapies, most research has historically been focused on insulin and the immune system. The identification of small molecules that modulate α-cell function constitutes a distinct strategy that may lead to useful candidate compounds. Potent and safe new drugs that specifically reduce glucagon secretion from α-cells without impacting other cells in the body could represent a major advance for both new-onset as well as established T1D (10). Based on results from the late Roger Unger and others, suppressing glucagon secretion can improve glucose control and reduce the need for insulin injections in rodent models (11).

Previously, a high-throughput screen of 200,000 compounds was completed to identify suppressors of glucagon production using the hamster InR1G9 α-cell line (12). 417 potential glucagon suppressors were discovered, three of which were determined to act in part by suppressing glucagon gene transcription, while the remaining compounds act by unknown mechanisms. To date, none of the hits had been validated in human islets. Here, we show that at least one class of these small molecules discovered in a the rodent α-cell screen is able to suppress glucagon release from human pancreatic islets without major impacts on insulin production. The results reported here provide information on chemical tools which may be used to extract new knowledge about the regulation of glucagon production and secretion from pancreatic islet α-cells.

## 2 Materials and methods

### 2.1 Reagents

Complete source information for reagents, chemicals, and antibodies used in this work are listed in **Supplementary Material** **Table S1**. Screening compounds were cherry-picked in the UT Southwestern High-Throughput Screening Core facility. The top 200 hits from the primary screen were selected according to multiple criteria. Effect size on glucagon release (RZ score > 1.48); low toxicity (< 10%); absence of non-specific effects on general secretory pathway (Gaussia RZ score > -3). Finally, 17 hits were eliminated because of benzimidazole structures known to be false positives in the primary screen (12). In all experiments where islet-conditioned CMRL medium is assayed for glucagon or insulin, phenol red-free CMRL was used to avoid interference in the HTRF assay.

### 2.2 Human islet receipt and culture

Cadaveric human islets were obtained through the Integrated Islet Distribution Program (IIDP). Islets were isolated by the affiliated islet isolation center and cultured in PIM medium (PIM-R001GMP, Prodo Labs) supplemented with glutamine/glutathione (PIM-G001GMP, Prodo Labs), 5% Human AB serum (100512, Gemini Bio Products), and ciprofloxacin (61-277RG, Cellgro, Inc) at 37°C and 5% CO2 until shipping at 4°C overnight. Human islets were cultured upon receipt in complete phenol red-containing CMRL-1066 (containing 1 g/L (5.5 mM) glucose, 10% FBS, 100 U/ml penicillin, 100 μg/ml streptomycin, 292 µg/mL L-glutamine). For testing compounds in intact human islets, 50-75 islets were hand-picked under a dissection microscope and transferred to low-binding 1.5 ml tubes Islets were exposed to compounds for 24 h in complete CMRL (phenol red-free) according to the legend. Medium supernatants were collected, centrifuged (10,000 x g; 5 min) and transferred to fresh tubes for storage at -80°C. Glucagon in the sample was measured using the Cisbio Glucagon HTRF kit. Total insulin and glucagon content was extracted by acid-ethanol (1.5% HCl in 70% ethanol) overnight at -80°C and the solution was neutralized with an equal volume of 1 M Tris pH 7.4 prior to insulin and glucagon HTRF assays. For studies of static culture stimulation of glucagon secretion, 50-75 islets were hand-picked and treated similarly as above. After the 24 h exposure to treatments, islets were treated based on an established paradigm (13). Briefly, islets were washed twice with 500 µL of KRBH (134 mM NaCl, 4.8 mM KCl, 1 mM CaCl_2_, 1.2 mM MgSO_4_, 1.2 mM KH_2_PO_4_, 5 mM NaHCO_3_, 10 mM HEPES pH 7.4, 0.1% radioimmunoassay-grade BSA) and incubated in 500 µL of KRBH with 5.5 mM glucose for 0.5 h, then 500µL KRBH containing 16 mM glucose for 1 h, and finally 500 µL KRBH containing 1 mM glucose with or without 1 µM epinephrine. Supernatants and islet lysates were collected, centrifuged (10,000 x g; 5 min) and transferred to fresh tubes for storage at -80°C. Glucagon in supernatants and lysates was measured by HTRF and secreted glucagon was normalized to glucagon content. Human islet characteristics and donor information are listed in **Supplementary Table S2** Human Islet Checklist.

### 2.3 Human islet dissociation, replating, and compound screening

To dissociate and replate human islet cells in 384-well format, we adapted a protocol developed by the Wagner lab at the Broad (14). Briefly, we utilized a thin coating of commercial growth factor-reduced extracellular matrix (Cultrex Reduced Growth Factor Basement Membrane Extract, Type 2, Pathclear) to coat plates for adherence of dissociated human islet cells and further compound testing. Briefly, Cultrex (kept cold on ice) was diluted 1:20 into cold phenol red-free CMRL medium and 25 µl of the mixture was dispensed to in black-walled, clear-bottom 384-well plates using a BioTek Multiflo. Plates were incubated at 37°C for at least 30 min to allow the ECM to solidify and coat the wells. 10,000 to 15,000 human islet equivalents (IEQs) were dissociated with StemPro Accutase for 20 min in a 37°C water bath, triturated 5-7 times, resuspended in complete phenol red-free CMRL-1066 medium (containing 1 g/L (5.5 mM) glucose, 10% FBS, 100 U/ml penicillin, 100 μg/ml streptomycin, 292 µg/mL L-glutamine), pelleted at 200 x g for 5 min, resuspended in complete phenol red-free CMRL medium, then passed through a 100 µm cell strainer and counted. Cells were diluted to 2.5E5 cells/mL and 1E4 cells were dispensed per well in a 40 µL volume using a BioTek Multiflo. Plates were incubated at 37°C overnight to allow cell attachment. On the following day, medium was exchanged for medium containing test compounds using a BioMek. In each trial, compounds were tested in triplicate. Cells were incubated for 24 h in the presence of compounds and medium was collected the following day using the BioMek to transfer samples to an opaque white 384-well plate. Glucagon concentrations of all triplicate treated samples were subsequently measured using the Glucagon HTRF assay.

### 2.4 Immunocytochemistry and confocal microscopy

Clear-bottom 384-well plates with human islet cells attached were fixed and permeabilized in 4% paraformaldehyde and 0.1% Triton X-100 in PBS (137 mM NaCl, 2.7 mM KCl, 10 mM Na2HPO4, 1.8 mM KH2PO4, pH 7.4) for 10 min at room temperature. Wells were washed three times with PBS for 5 min each and then blocked for 1 h in normal donkey serum (NDS) blocking buffer (2% donkey serum, 1% bovine serum albumin, 0.1% cold fish skin gelatin, 0.1% Triton X-100, 0.05% sodium azide, 0.05% Tween-20, PBS). Cells were incubated overnight at 4°C in NDS buffer containing primary antibodies (indicated in **Table S1**), followed by three 10 min washes in PBS, incubation in corresponding dye-linked secondary antibodies and DAPI in NDS buffer, and a final three 10 min washes in PBS. Stained cells were imaged on a Zeiss LSM 700 AxioObserver equipped with a Plan-Apochromat 20x/0.8 M27 objective.

### 2.5 Glucagon and insulin measurements

Glucagon and insulin were measured using homogenous time-resolved FRET (HTRF) assays (Cisbio/PerkinElmer). Glucagon HTRF assays in the high-throughput screening core facility were read on a CLARIOstar multimode plate reader (BMG Labtech). Glucagon HTRF assays for intact islet static culture samples were run on a PheraStar FS (BMG Labtech). Before measuring glucagon, islet lysates and medium supernatants were diluted in 1X diluent #5 (per HTRF instructions) to achieve results within the standard curve of the assay. Dilution factors were determined empirically. Islet lysates typically diluted 400- to 500-fold, medium 20- to 100-fold, and KRBH samples from 1 h static culture were not diluted.

### 2.6 Relative gene expression

RNA was isolated from intact human islets using Quick-RNA Microprep kit (Zymo). Briefly, after indicated treatments, medium was removed from islets and RNA lysis buffer (with β-mercaptoethanol) was added to cells in 1.5 mL tubes, vortexed, and then transferred to -80°C for storage. Samples were processed according to the kit manufacturer’s instructions, including on-column digestion with RNase-free DNase. RNA concentration was measured using a Nanodrop spectrophotometer and verified to have A_260/280_ ratios >2.0. 600 ng of RNA was converted into cDNA using the iScript cDNA synthesis kit (Bio-Rad) following manufacturer instructions and the resulting cDNA was diluted 10-fold with water. One μl of diluted cDNA was used in 10 μl qPCR reactions using 2X SYBR Bio-Rad master mix and 250 nM of each primer. Reactions were run in 384-well format on a CFX384 (Bio-Rad) or QuantStudio 5 (Thermo). qPCR data was analyzed using CFX Manager (Bio-Rad) with 18S RNA as the reference gene. Relative expression was calculated by the 2^-ΔΔCt^ method.

### 2.7 InsGLuc-MIN6 β-cell insulin secretion reporter counter-screen

Culture of MIN6 β-cells (RRID:CVCL_0431) has been described (15). MIN6 cells are cultured in complete medium (DMEM containing 4.5 g/L (25 mM) glucose, 292 µg/L glutamine, 10% FBS, 50 µM β-mercaptoethanol, 1 mM pyruvate, 100 U/ml penicillin, and 100 μg/ml streptomycin). InsGLuc-MIN6 β-cells and their use in high-throughput screening has been described (15, 16). Briefly, cells were seeded at ~15e6 cells per T175 flask and grown 7 days until confluence, then trypsinized, resuspended in complete medium, passed through a 40 µm cell strainer and diluted to 1.5e6 cells/ml. Cells were dispensed at 50 µl per well in 384-well opaque white cell culture dishes using a BioTek Multiflo FX liquid handler to yield 7.5e4 cells/well. Plates were placed in a tissue culture incubator for 72 h. Next, 0.5 µl each of DMSO (negative control, 1%), thapsigargin (positive control, 1 mM), and small molecules (5 µM) were added robotically to the plates and the cells were incubated 24 h. On the day of the assay, using a BioTek Multiflo FX liquid handler, cells were washed twice with KRBH (75 µL then 50 µL). To remove medium and buffer between washes, plates were centrifuged upside-down in collection trays at 30 x g for 1 min. Cells were then incubated in 25 µl of KRBH containing 200 mM diazoxide for 60 min. Next, 25 µl of 2X stimulation buffer in KBRH was added to yield a final concentration of 20 mM glucose, 35 mM KCl and 250 mM diazoxide in a total volume of 50 µl. The plates were incubated for 60 min at 37°C in a tissue culture incubator. Afterward, 20 µl of GLuc assay working solution (5 mM KCl, 15 mM Hepes pH 7.4, 24 mM NaHCO_3_, 1 mM MgCl_2_, 2 mM CaCl_2_, 300 mM sodium ascorbate, and 3.54 µM coelenterazine) was added to each well. Within 10-20 min, the luminescence from each well of the plates was measured using a Perkin Elmer EnVision multi-mode plate reader with 0.1 sec integration time. The raw data were corrected for plate effects using a GeneData proprietary algorithm and then each value on a per plate basis was subjected to either single-point or two-point normalization, followed by calculation of the condensed activity and robust Z (RZ) score for each well.

### 2.8 Statistics, data analysis, and figure preparation

**Figure 1:**
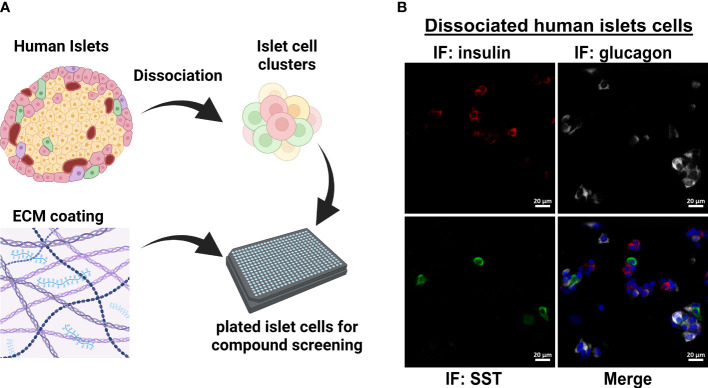
Workflow for dissociated human islet assay for screening glucagon release inhibitors. **(A)** Workflow for dissociating and replating human islets into 384-well format for compound testing. **(B)** Dissociation and replating of human islet cells successfully represents the three major islet cell types in a 384-well format for HTS assays.

Quantitated data are expressed as mean ± SE where indicated. Two-way ANOVA with indicated post-hoc test was used for data with more than one variable, otherwise Student’s t-test was used, and considered significant if P<0.05. Graphs were made in GraphPad Prism 9 and figures assembled in Affinity Designer (Serif). Cartoon model in **Figure 1A** was created using BioRender.

## 3 Results

### 3.1 Human islet cell platform for testing small molecule effects on glucagon production

The original 200,000 compound primary screen used an Alpha-Screen assay to measure glucagon released into the medium after 24 h from the InR1G9 hamster α-cell line (12). The primary aim of the current work was to determine the potential translatability of these candidates from rodent cell culture to human islets. To validate small molecule hits in human islets, we adapted a previously successful protocol for dissociating and plating human islet cells into 384-well format (**Figure 1A**) (17). We established that after replating the dissociated islets, the three major islet endocrine cell types were represented: insulin-producing β-cells, glucagon-producing α-cells, and somatostatin-producing δ-cells (**Figure 1B**). For human islet cell validation studies, only the most active and repeatable compounds from the primary screen were selected. Starting with the 957 primary hits from Evans, et al (12), we selected compounds with a robust Z-score >3, <10% toxicity, and <10% effect on the constitutive secretory pathway. In addition, we eliminated 17 compounds known to have a benzimidazole structure that interfered with the assay in the primary screen (12). These filtering steps resulted in 200 small molecules to be re-picked from the high-throughput screening core library.

### 3.2 Retesting top candidate glucagon secretion suppressors

**Figure 2:**
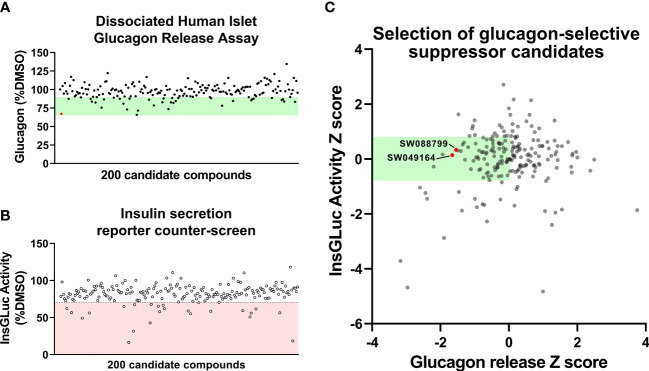
Glucagon release and insulin secretion reporter counter-screening in dissociated human islet assay. **(A)** HTS testing of candidate glucagon inhibitors in the dissociated human islet assay provided means to rank candidates. **(B)** Counter-screen of candidate compounds in an insulin secretion reporter assay [InsGLuc-MIN6 (15, 16)]. **(C)** Glucagon release data and InsGLuc activity data were converted to Z scores and plotted against one another to select glucagon secretion suppressor compounds with little impact on β-cell function. Data represent the mean of screening assays from 3-4 independent donor islet preparations.

To identify compounds that exhibit effects in human α-cells, we replated human islets cells into 384-well format as in **Figure 1B** and exposed them to candidate glucagon suppressor compounds for 24 h in complete phenol red-free CMRL-1066 which contained 5.5 mM glucose. The screen was repeated three times using islets from three independent donors (**Table S2**). Following this chronic exposure, medium was collected and glucagon concentration was measured using a homogenous time-resolved fluorescence (HTRF) assay. Compared to vehicle treatment, a hit compound from the original screen [SW088811 (12)] reduced glucagon concentrations in the medium by ~33% (**Figure 2A**). Most compounds were less potent than the positive control, but caused between 10% to 30% suppression of glucagon release.

To determine candidate compounds with selective effects on glucagon, we counter-screened the candidates in our insulin secretion reporter assay, InsGLuc-MIN6 β-cells (15, 18). This reporter assay works using optimized Gaussia luciferase (GLuc) (19) inserted within the C-peptide region of insulin and its expression driven by the rat insulin promoter to generate InsGLuc. This biosensor was used to create stable the InsGLuc-MIN6 β-cell line, now a well-established strategy (16, 18, 20–23). After 24 h exposure of InsGLuc-MIN6 cells to candidate compounds, glucose-responsive insulin secretion was assessed (**Figure 2B**). Compounds which altered activity in the assay by < 30% were retained. The glucagon and insulin secretion data were cross-referenced to select compounds with relatively little impact in the insulin secretion reporter assay but which caused suppression of glucagon release in our human islet assay (**Figure 2C**). From the candidates yielded by this analysis, available compounds were restocked for further testing in intact islets.

### 3.3 Chronic exposure of intact human islets to SW088799 suppressed glucagon release

**Figure 3:**
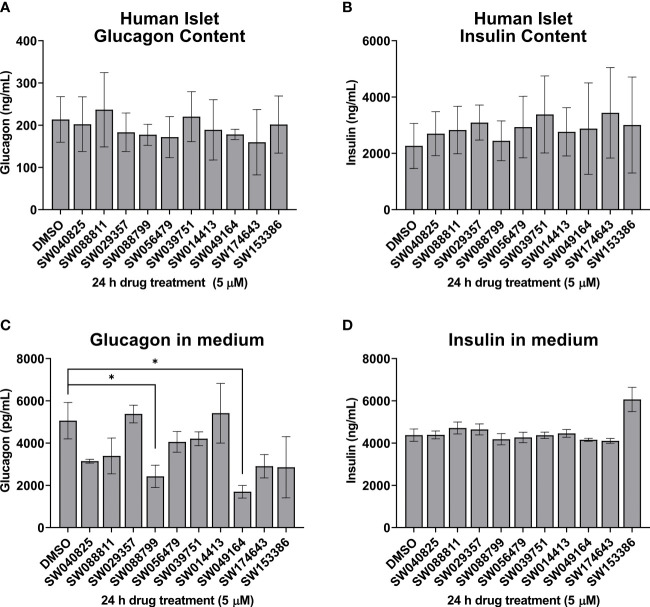
Hits from human islet validation screen retested in batch static incubation. Human islets were hand-picked (50 per 1.5mL tube) and incubated 24h in complete CMRL-1066 in the presence of DMSO (0.1%) or test compounds (5 µM). Glucagon content **(A)** and accumulation in the medium **(B)** are shown. Insulin content **(C)** and accumulation in the medium **(D)** are shown. Data represent the mean ± SE of 2-3 independent batches of human islets. *, P<0.05 by two-way ANOVA with Dunnett’s multiple comparisons test.

**Figure 4:**
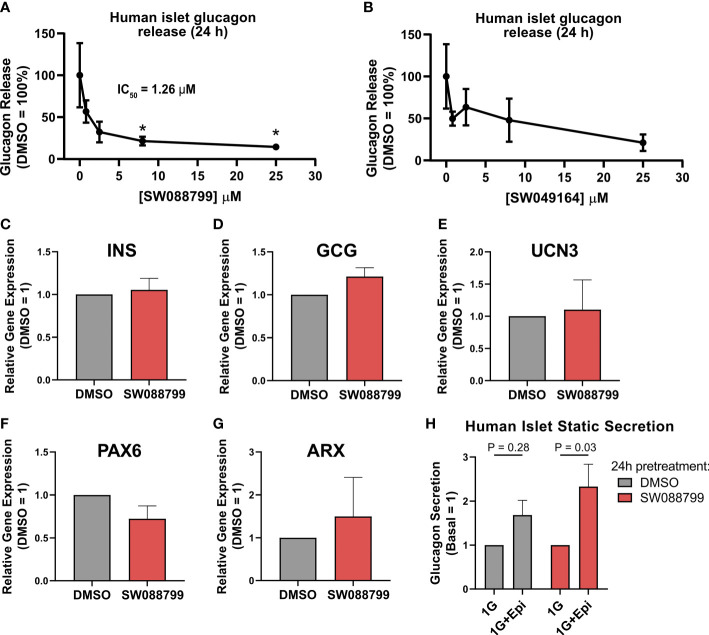
Impacts on glucagon release and islet gene expression in intact islets exposed to SW088799. **(A,B)** 50 human islets were treated in complete CMRL-1066 with vehicle (DMSO 0.1%) or indicated concentration of **(A)** SW088799 or **(B)** SW049164 (0.8, 2.5, 8, 25 µM) for 24 h Concentration of glucagon in medium was normalized to the total islet glucagon content and plotted as percent of DMSO. Data represent the mean ± SE of three independent experiments. *, P<0.05 vs DMSO by two-way ANOVA with Dunnett’s multiple comparisons test. **(C-G)** Expression of *INS*, *GCG*, *UCN3*, *PAX6*, and *ARX* were measured by qPCR from intact human islets were exposed to DMSO (0.1%) or SW88799 (10 µM) for 24 h Data are the mean ± SE from N=3 batches of donor islets. **(H)** 50 human islets were exposed to DMSO (0.1%) or SW088799 (10 µM) for 24 h before washout in KRBH (5.5mM glucose 30min, 16mM glucose 1h) followed by incubation in 1mM glucose KRBH with or without epinephrine (1 µM) for 1 h Data are the mean ± SD from N=3 batches of donor islets.

We next assessed the ability of the selected candidate compounds to chronically modulate the content and accumulation in the medium of insulin and glucagon from intact human islets. Islets were incubated in complete medium in the presence or absence of test compounds (5 µM) for 24 h. Afterward, medium and islet lysates were collected to measure concentrations of islet hormones. Overall, the compounds had little impact on insulin or glucagon content (**Figures 3A, B**). However, SW088799 and SW049164 reduced glucagon accumulation in the medium without impacting insulin accumulation (**Figures 3C, D**), confirming our rescreening results in dissociated human islet cells (**Figure 2**). One compound, SW153386, appeared to increase insulin in the medium, although we did not detect acute insulin secretory activity for this compound in our InsGLuc-MIN6 counter-screen. To determine potency of the two hit glucagon suppressors, SW088799 and SW049164, we performed concentration-response curves in intact human islets treated with compounds for 24 h. Glucagon concentration in the medium declined with increasing concentration of SW088799 (**Figure 4A**) and SW049164 (**Figure 4B**), however only SW088799 exhibited statistically significant effects with an apparent IC_50_ of 1.26 µM.

To determine whether SW088799 impacted key islet genes, we exposed intact islets to vehicle or SW88799 for 24 h and isolated RNA for qPCR. SW088799 did not significantly impact expression of INS (P = 0.73), *GCG* (P=0.17), *UCN3* (P=0.84), *PAX6* (P=0.21), or *ARX* (P=0.64) (**Figures 4C-G**). We also tested whether acute stimulation of glucagon release via adrenergic stimulation was affected after 24 h exposure to SW088799. Human islets exposed to either vehicle or SW088799 for 24 h each secreted glucagon in response to epinephrine after a brief 1.5 h washout period in KRBH (**Figure 4H**). These results suggested that SW088799 did not continue to suppress acute secretory responses after withdrawal of drug treatment.

### 3.4 Structural analysis of SW088799 to guide future mechanism of action studies

**Figure 5:**
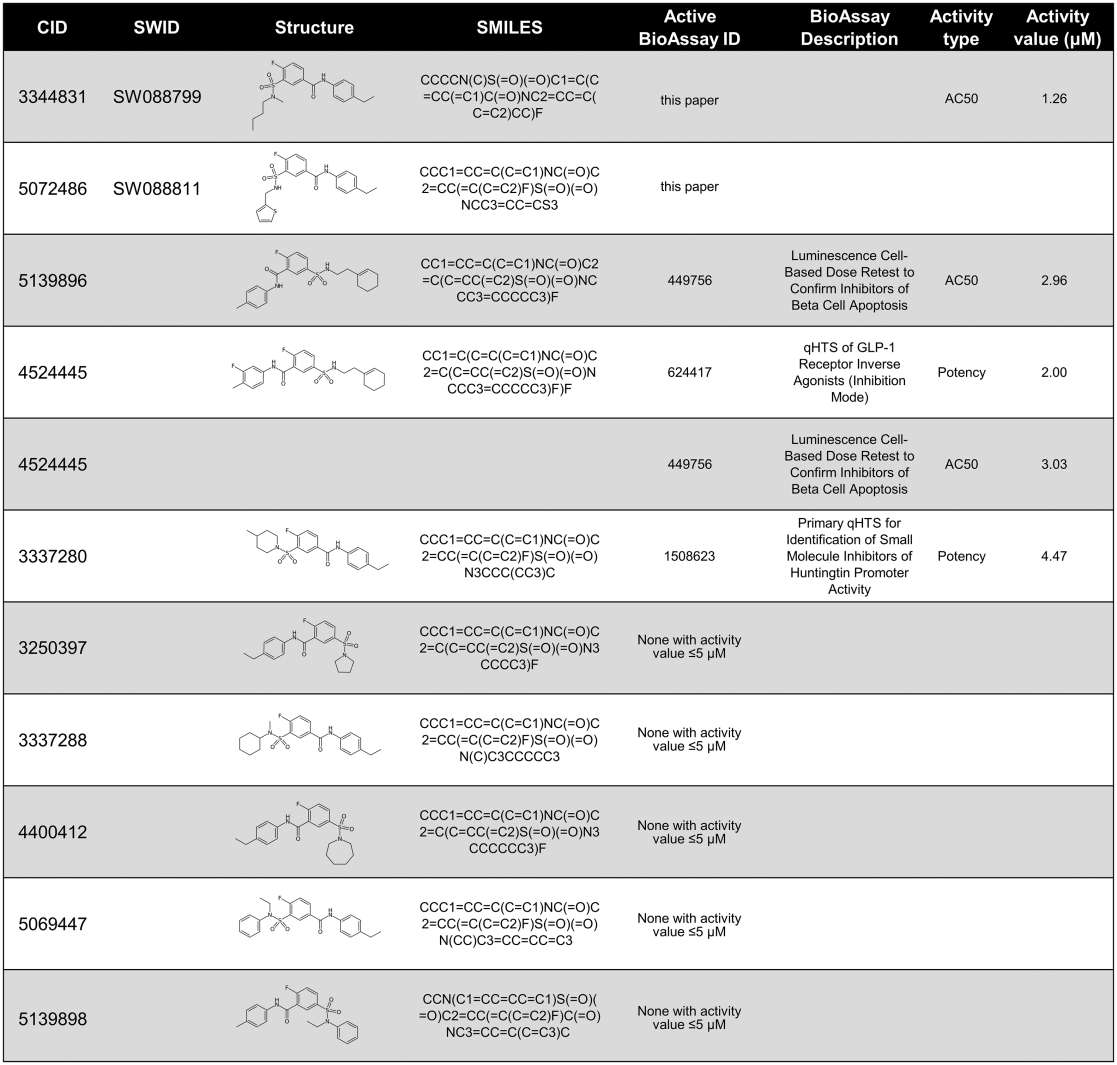
PubChem data on compounds structurally-related to SW088799. Compounds are listed by PubChem CID and 2D structures shown with SMILES notation. Compounds tested in this current work, SW088799, and in previous work, SW088811 (12), are denoted by their UT Southwestern identifier (SWID). PubChem was searched on November 18, 2022 to identify compounds with a Tanimoto similarity score of at least 97% to SW088799 which had also been reported in PubChem BioAssays. If a compound had activity in an associated PubChem BioAssay with ≤ 5 µM, the BioAssay ID and descriptions are shown, as well as the type of activity measurement and the value in µM.

The IUPAC name for SW088799 is 3-[butyl(methyl)sulfamoyl]-N-(4-ethylphenyl)-4-fluorobenzamide. Interestingly, one of the top hits in the primary InR1G9 α-cell screen (12), SW088811, is also in the fluorobenzamide class and is structurally similar to SW088799 (**Figure 5**), suggesting a similar mode of action even though SW088811 had less activity in intact human islets (**Figure 3C**). A PubChem search for compounds with 97% Tanimoto similarity with the fluorobenzamide SW088799 (CID 3344831) resulted in 196 compounds. 59 of these have been reported as tested in BioAssays in the PubChem database, eight of which were active and only four of those had activity values ≤ 5 µM (**Figure 5**). Two of these hits (CID 5139896 and CID 4524445) had protective effects in a viability screen using rat INS1E cells exposed to proinflammatory cytokines (TNFα, IFNγ, IL1β, 48 h) (24), although they were not top hits in that screen. CID 4524445 was also active as an inverse agonist of GLP1R (25). While the exact molecular mechanisms and protein targets are not known, the activities of some of these fluorobenzamides and related structures imply that future development could yield useful molecular tools for modulating pancreatic islet cell phenotypes.

## 4 Discussion

Understanding glucagon production and secretion is critical because α-cell dysfunction contributes to unstable blood glucose concentrations in both T1D and T2D (26–28), compounding the pathology of these diseases. Here we demonstrated the feasibility of translating an α-cell line-based high-throughput screen into a primary dissociated human islet system for glucagon suppressor validation. Using this approach, we successfully confirmed that fluorobenzamide compounds, a class first identified in the previously published cell-based screen (12), also suppress glucagon release from dissociated and intact human islet cells *in vitro*. As the exact molecular mechanism(s) of these compounds in α-cells is not currently understood, further testing and structure-activity relationship studies are still necessary to address these questions and generate tool compounds for target identification. Previous work indicated that in addition to reducing glucagon release, the fluorobenzamide SW088811 suppressed *Gcg* gene expression in InR1G9 α-cells (12). Given little impact was observed on glucagon content with either SW088811 or SW088799, it is unclear if reduced *GCG* expression in human islets is the primary mechanism of suppressed glucagon release. Indeed, we did not observe a significant effect on expression of *GCG*, or on transcription factors that affect α-cell function including *ARX* and *PAX6* (29, 30). We also did not observe significant impact on expression of *INS* or the β-cell maturation marker *UCN3*, suggesting against effects on islet cell differentiation status. Alternatively, because α-cells can be regulated in a paracrine manner within the islet, it is possible some of these compounds are indirect modulators of glucagon production through impacting other islet cell types. Islet δ-cells secrete somatostatin in response to glucose, but also require β-cell-secreted urocortin 3 for full activity (31). There is also evidence of δ-cell-β-cell cross-talk through gap junctions to induce somatostatin secretion and suppress glucagon secretion (32). Glucagon signaling on the β-cell can also augment insulin secretion and is therefore raises an issue pertaining to suppressing glucagon. However, the potential loss of a glucagon-mediated potentiating effect on remaining β-cells due to a reduction in basal glucagon release may be less critical than a beneficial reduction in hyperglycemia induced by suppression of α-cell glucagon secretion.

We found it interesting that the SW088799-related compound CID 4524445 was active in a high-throughput assay for glucagon-like peptide 1 (GLP1) receptor (GLP1R) inverse agonism (**Figure 5**) (25). GLP1R has been shown to be expressed in mouse and human α-cells and impact glucagon secretion in a glucose-dependent manner (33). Islets from mice with α-cell-specific deletion of GLP1R had lower glucagon secretion at low glucose and higher glucagon secretion at high glucose compared to wild-type islets. Additional phenotypic assays are required focused on SW088799 and similar compounds to determine whether they may act through GLP1R or a related pathway to impact α-cell function. Along these lines, new α-cell targets are continuing to be identified which have potential for small molecule interventions. Recent work showed the olfactory marker protein (*Omp*) regulates cAMP in α-cells and affects AMP kinase activity (34). α-cell-specific *Omp* knockout mice have reduced baseline AMPK phosphorylation and knockdown of *Omp* in an α-cell line caused a loss of glucose-induced reduction in cAMP levels. Continued exploration of α-cell function regulators through phenotypic or genetic screening approaches has the potential to uncover more therapeutic pathways and tool compounds.

A related idea to regulate glucagon signaling is the disruption of glucagon receptor activation either genetically or through use of injected antibodies. While this can result in α-cell hyperplasia (35, 36), this effect may offer a potential source of new β-cells if they can be transdifferentiated. Indeed, beneficial effects have been observed for injected anti-glucagon receptor antibodies in humans with T1D (7) as well as non-human primates (4, 8) and rodents (9). Other recent studies in mice have suggested that a combination of α-to-β-cell transdifferentiation and β-cell replication can occur in response to glucagon receptor antagonist antibodies (4, 5, 37). In the future, an ideal α-cell-targeted therapeutic would suppress glucagon secretion selectively under hyperglycemia, but permit secretion during hypoglycemia. Additionally, treatments with the ability to avoid unintended α-cell hyperplasia may be desirable.

## 5 Limitations of the study

Dissociation of human islets into single cells may alter the secretory responses of α-cells (38, 39). To mitigate such effects, we dissociated islets into clusters according to the protocol of Walpita et al. (14) (**Figure 1B**), and avoid dissociation to the single-cell level. Another limitation is that our screening conditions were in CMRL-1066 which contains a relatively physiological 5.5 mM glucose concentration, as well as serum, amino acids, and other additives. In T1D, α-cells may lose their ability to suppress glucagon under hyperglycemic conditions. However, because our screen used healthy human islets, normal CMRL-1066 was chosen to maintain cell viability in multiple days of culture while also allowing the α-cells to release glucagon. Under these conditions, hit compounds that suppress α-cell function would be detectable. Finally, these data are derived from static culture incubation experiments and more detailed dynamic secretion studies will be necessary in the future to better understand the pharmacological impact on basal and stimulated glucagon secretion.

## Data availability statement

The raw data supporting the conclusions of this article will be made available by the authors, without undue reservation.

## Author contributions

MK conceptualized, administered/supervised, and executed the studies, analyzed data, acquired funding, and wrote and edited the manuscript. KR, DB, and SW executed studies and helped with methodology. KR and AZ analyzed data. ME and MR provided resources. BP and MC acquired funding and provided supervision. All authors contributed to the article and approved the submitted version.

## Supplementary material

Supplementary Table 1 Reagents and Antibodies.

Supplementary Table 2 Human Islet Donor Checklist
