# Supplementary material for: Small molecule glucagon release inhibitors with activity in human islets
Source: Front Endocrinol (Lausanne). 2023 Apr 19;14:1114799. doi: 10.3389/fendo.2023.1114799 (PMC10157210; doi:10.3389/fendo.2023.1114799)
Supplement: Supplementary Table 1 — Reagents and Antibodies. [file Table_1.pdf]

Table S1. Reagents and Antibodies

Reagents

Name	Company	Catalog#
SW088799	ChemDiv	K781-6672
SW049164	Chembridge	7349063
SW029357	ChemDiv	4120-0156
SW056479	ChemDiv	8009-8144
SW039751	ChemDiv	4896-4668
SW014413	ChemDiv	3011-0242
SW174643	Chembridge	7451148
SW153386	Chembridge	5580829
SW026536	VitasM	STL086053
SW027198	VitasM	STK831336
SW040825	Interbioscreen	STOCK3S-50841
SW088811	ChemDiv	K781-6706
Cultrex Reduced Growth Factor Basement Membrane Extract, Type 2, Pathclear	R&D	3533-010-02
Insulin HTRF	Cisbio / Perkin Elmer	62IN1PEG
Glucagon HTRF	Cisbio / Perkin Elmer	62CGLPEG
Pen/Strep/Gln, 100X	Corning	30009CI
CMRL-1066	ThermoFisher	11530037
CMRL-1066 Phenol red-free	Corning	99-603-CV

Antibodies

Protein	Host Species	Company	Cat#	Dilution
Insulin	guinea pig	Abcam	ab7842	1:100
Glucagon	mouse	Sigma	G2654	1:5000
Somatostatin	goat	Santa Cruz	sc-7819	1:50
anti-guinea pig Cy3 (555)	donkey	Jackson Immuno	706165148	1:400
anti-mouse 647	donkey	ThermoFisher Scientific	A31571	1:400
anti-goat 488	donkey	ThermoFisher Scientific	A-11055	1:400
DAPI	n/a	ThermoFisher Scientific	D1306	300 nM
