# Supplementary material for: Small molecule glucagon release inhibitors with activity in human islets
Source: Front Endocrinol (Lausanne). 2023 Apr 19;14:1114799. doi: 10.3389/fendo.2023.1114799 (PMC10157210; doi:10.3389/fendo.2023.1114799)
Supplement: Supplementary Table 2 — Human Islet Donor Checklist [file Table_2.pdf]

Checklist for reporting human islet preparations used in research

Adapted from Hart NJ, Powers AC (2018) Progress, challenges, and suggestions for using human islets to understand islet biology and human diabetes. Diabetologia https://doi.org/10.1007/s00125-018-4772-2

Islet preparation	1	2	3	4	5	6	7	8[a]
**MANDATORY INFORMATION**								
Unique identifier	SAMN11250012	SAMN11244711	SAMN11864195	SAMN12129273	SAMN12333862	SAMN12496804	R355	SAMN13836615
Donor age (years)	56	56	53	30	24	40	79	58
Donor sex (M/F)	F	F	F	F	M	F	M	M
Donor BMI (kg/m^2)	29.2	39.5	40	35.7	23.9	30.4	23.5	23.2
Donor HbA_{1c}	5.2%	5.8%	5.5%	4.5%	5.0%	5.0%	5.2%	5.7%
Origin/source of islets[b]	IIDP	IIDP	IIDP	IIDP	IIDP	IIDP	UAB	IIDP
Islet isolation centre	University of Miami	University of Wisconsin	University of Wisconsin	SoCal-ICRC	University of Pennsylvania	University of Miami	University of Alaabam	The Scharp-Lacy Research Institute
Donor history of diabetes? Please select yes/no from drop down list	No	No	No	No	No	No	No	No
**If Yes, complete the next two lines if this information is available**								
Diabetes duration (years)								
Glucose-lowering therapy at time of death[c]								
**RECOMMENDED INFORMATION**								

Donor cause of death	cerebrovascular/ stroke	cerebrovascular/ stroke	cerebrovascular/ stroke	anoxia	anoxia	cerebrovascular/ stroke	n/a	cerebrovascular/ stroke
Warm ischaemia time (min)	5	no	no	12	no	no	n/a	no
Cold ischaemia time (min)	557	570	403	636	1083	510	n/a	452
Estimated purity (%)	90	94	92	90	95	90	80	90
Estimated viability (%)	94	99	98	96	93	92	n/a	95
Total culture time (h)[d]	72h	64h	72h	50h	49h	96h	62h	92h
Glucose-stimulated insulin secretion stimulation index (SI) by IIDP	1.9	5.3	2.4	1.8	1.6	1	n/a	4.9
Handpicked to purity? Please select yes/no from drop down list	Yes	Yes	No	No	No	No	Yes	Yes
Additional notes	Fig 1B	Fig 1B	Fig 2B	Fig 2B	Fig 2B	Fig 2B	Fig 4AB	Fig 4AB

[a]If you have used more than eight islet preparations, please complete additional forms as necessary
[b]For example, IIDP, ECIT, Alberta IsletCore
[c]Please specify the therapy/therapies
[d]Time of islet culture at the isolation centre, during shipment and at the receiving laboratory
[e]Please specify the test and the results

Islet preparation	9	10	11	12	13	14	15	16[a]
**MANDATORY INFORMATION**								
Unique identifier	SAMN13881228	SAMN13175912	SAMN13221385	SAMN13515839	SAMN13739565	SAMN10536140	HP-22362-01	SAMN32641505
Donor age (years)	34	34	54	64	42	58	51	16
Donor sex (M/F)	M	F	F	M	M	F	M	M
Donor BMI (kg/m^2)	28.1	30.3	31.7	20.9	37.3	30	24.9	29.5
Donor HbA_{1c}	5.6%	4.8%	5.0%	5.5%	5.6%	5.8%	5.3%	5.4%
Origin/source of islets[b]	IIDP	IIDP	IIDP	IIDP	IIDP	IIDP	Prodo	IIDP
Islet isolation centre	SoCal-ICRC	University of Pennsylvania	University of Miami	The Scharp-Lacy Research Institute	The Scharp-Lacy Research Institute	SoCal-ICRC	Prodo	SoCal-ICRC
Donor history of diabetes? Please select yes/no from drop down list	No	No	No	No	No	No	No	No
**If Yes, complete the next two lines if this information is available**								
Diabetes duration (years)								
Glucose-lowering therapy at time of death[c]								
**RECOMMENDED INFORMATION**								
Donor cause of death	head trauma	anoxia	head trauma	cerebrovascular/ stroke	anoxia	anoxia	cerebrovascular/ stroke	head trauma

Warm ischaemia time (min)	10	30	no	no	11	16	N/A	no
Cold ischaemia time (min)	534	855	750	515	431	595	N/A	372
Estimated purity (%)	85	95	80	95	90	80	85	80
Estimated viability (%)	95	93	88	95	95	95	95	96
Total culture time (h)[d]	87	159	96	72	90	72	N/A	53
Glucose-stimulated insulin secretion stimulation index (SI) by IIDP	0.5	1.4	3.4	3.6	6.7	3.5	N/A	2.4
Handpicked to purity? Please select yes/no from drop down list	Yes	Yes	Yes	Yes	Yes	Yes	Yes	Yes
Additional notes	Fig 4AB	Fig 3	Fig 3	Fig 3	Fig 4AB	Fig 1B	Fig 4C-G	Fig 4C-G

[a]If you have used more than eight islet preparations, please complete additional forms as necessary
[b]For example, IIDP, ECIT, Alberta IsletCore
[c]Please specify the therapy/therapies
[d]Time of islet culture at the isolation centre, during shipment and at the receiving laboratory
[e]Please specify the test and the results

Islet preparation	17	18	19	20	21	22	23	24[a]
**MANDATORY INFORMATION**								
Unique identifier	HP-23019/20-01	HP-23044-01	HP-23052-01	SAMN33826313				
Donor age (years)	55	36	63	28				
Donor sex (M/F)	M	F	M	M				
Donor BMI (kg/m^2)	30.4	34	22.1	29.3				
Donor HbA_{1c}	5.6%	4.9%	5.2%	5.0%				
Origin/source of islets[b]	Prodo	Prodo	Prodo	IIDP				
Islet isolation centre	Prodo	Prodo	Prodo	Imagine Pharma				
Donor history of diabetes? Please select yes/no from drop down list	No	No	No	No				
**If Yes, complete the next two lines if this information is available**								
Diabetes duration (years)								
Glucose-lowering therapy at time of death[c]								
**RECOMMENDED INFORMATION**								
Donor cause of death	anoxia	anoxia	cerebrovascular/ stroke	head trauma				
Warm ischaemia time (min)	N/A	N/A	N/A	N/A				
Cold ischaemia time (min)	N/A	N/A	N/A	N/A				

Estimated purity (%)	90	85	90	85				
Estimated viability (%)	95	95	95	87				
Total culture time (h)[d]	N/A	N/A	N/A	96				
Glucose-stimulated insulin secretion stimulation index (SI) by IIDP	N/A	N/A	N/A	N/A				
Handpicked to purity? Please select yes/no from drop down list	Yes	Yes	Yes	Yes				
Additional notes	Fig 4C-G	Fig 4H	Fig 4H	Fig 4H				

[a]If you have used more than eight islet preparations, please complete additional forms as necessary
[b]For example, IIDP, ECIT, Alberta IsletCore
[c]Please specify the therapy/therapies
[d]Time of islet culture at the isolation centre, during shipment and at the receiving laboratory
[e]Please specify the test and the results
